# Improving Anticoagulation Care for Pediatric Oncology Patients: A Quality Improvement Initiative

**DOI:** 10.1097/pq9.0000000000000720

**Published:** 2024-02-09

**Authors:** Vilmarie Rodriguez, Brockton S. Mitchell, Joseph Stanek, Katherine Vasko, Jean Giver, Kay Monda, Joan Canini, Amy A. Dunn, Riten Kumar

**Affiliations:** From the * Division of Hematology/Oncology/BMT, Nationwide Children’s Hospital, Columbus, Ohio; † Department of Pediatrics, The Ohio State University College of Medicine, Columbus, Ohio; ‡ Biostatistics Core, Nationwide Children’s Hospital, Columbus, Ohio; § Dana Farber/Boston Children’s Cancer and Blood Disorders Center, Boston, Mass.; ¶ Harvard Medical School, Boston, Mass.

## Abstract

**Background:**

Cancer is associated with increased venous thromboembolism in children. Risk factors for venous thromboembolism in this cohort include using central venous catheters, mass effect from underlying malignancy, chemotherapy, and surgery. Anticoagulation management in this cohort is challenging, given recurrent episodes of thrombocytopenia, the need for invasive procedures, and coagulopathy. A quality improvement (QI) initiative was developed to improve hematology consultation services and provide documentation of an individualized anticoagulation care plan for this high-risk cohort.

**Methods:**

Through the use of QI methods, interviews of stakeholders, expert consensus, and review of baseline data, a multidisciplinary team was organized, and key drivers relevant to improving access to hematology consultations and documentation of individualized anticoagulation care plans were identified. We used a Plan-Do-Study-Act model to improve hematology consultations and documentation of anticoagulation care plan (process measure). Outcome measures were bleeding and thrombosis recurrence/progression.

**Results:**

Seventeen patients with oncologic and venous thromboembolism diagnoses were included as baseline data. Slightly over half of these patients [53% (n = 9)] had a hematology consultation, and 7 (43.8%) had documentation of an anticoagulation care plan. After implementing QI methods, all 34 patients (100%) received hematology consultations and documentation of an anticoagulation care plan, and this measure was sustained for 1 year. Bleeding and thrombosis rates were similar in the baseline and post-QI cohorts.

**Conclusions:**

QI interventions proved effective in sustaining access to hematology consultations and providing anticoagulation care plans for patients with concomitant improved anticoagulation plan documentation for patients.

## INTRODUCTION

### Problem Description

Anticoagulation in patients with cancer is complicated by bleeding risk and venous thromboembolism (VTE) recurrence despite antithrombotic therapy.^1–4^ In children with an underlying malignancy, VTE has been reported in 2%–16% and is associated with increased mortality, thrombus recurrence, hemorrhage, loss of venous access, and postthrombotic syndrome.^5–9^ Anticoagulation therapy remains a challenge in this medically complex cohort due to their increased risk for bleeding (eg, thrombocytopenia, mucositis, and the need for invasive procedures).

### Available Knowledge and Rationale

CPGs standardize patient care, reduce practice variation, and promote efficient healthcare utilization. Several scientific societies and organizations, including the American College of Chest Physicians and the National Comprehensive Cancer Network have developed evidence-based guidelines on the diagnosis, treatment, and prevention of VTE in adult cancer patients.^2,3,10^ In stark contrast, there are no specific guidelines for treating VTE in pediatric oncology.

At Nationwide Children’s Hospital (NCH), pediatric hematology and oncology clinical services are separate, with the hematology service responsible for VTE consultation and antithrombotic therapy recommendations. Over the last few years, we noticed an increased incidence of VTE in our oncology population. Consequently, hematology involvement in the anticoagulation management of these patients, specifically around the use of direct oral anticoagulants (DOACs), peri-procedure bridging, and holding anticoagulation during periods of thrombocytopenia was needed.

### Specific Aims

To improve access to thrombosis and anticoagulation consultation and to standardize anticoagulation care plans for each patient, a multidisciplinary team was organized, and using QI methodology, interventions were developed to address the identified gaps. Our primary aim was to improve hematology consultation and anticoagulation care plan (process measure) documentation in oncology/BMT patients receiving anticoagulation therapy from a baseline of 12%–90% in 6 months and sustain it for 1 year. The secondary aim (outcome measures) was to reduce bleeding and thrombosis progression/recurrence in oncology/BMT patients receiving anticoagulation.

## METHODS

### Context

This report describes a QI study conducted at NCH, a quaternary pediatric teaching hospital in Columbus, Ohio, serving more than one million patients annually. Revised Standards for Quality Improvement Reporting Excellence (Squire 2.0) guided the study design, conduct, and writing of this manuscript.^11^ The project was deemed exempt from NCH institutional review board review and approval.

**Fig. 1.**
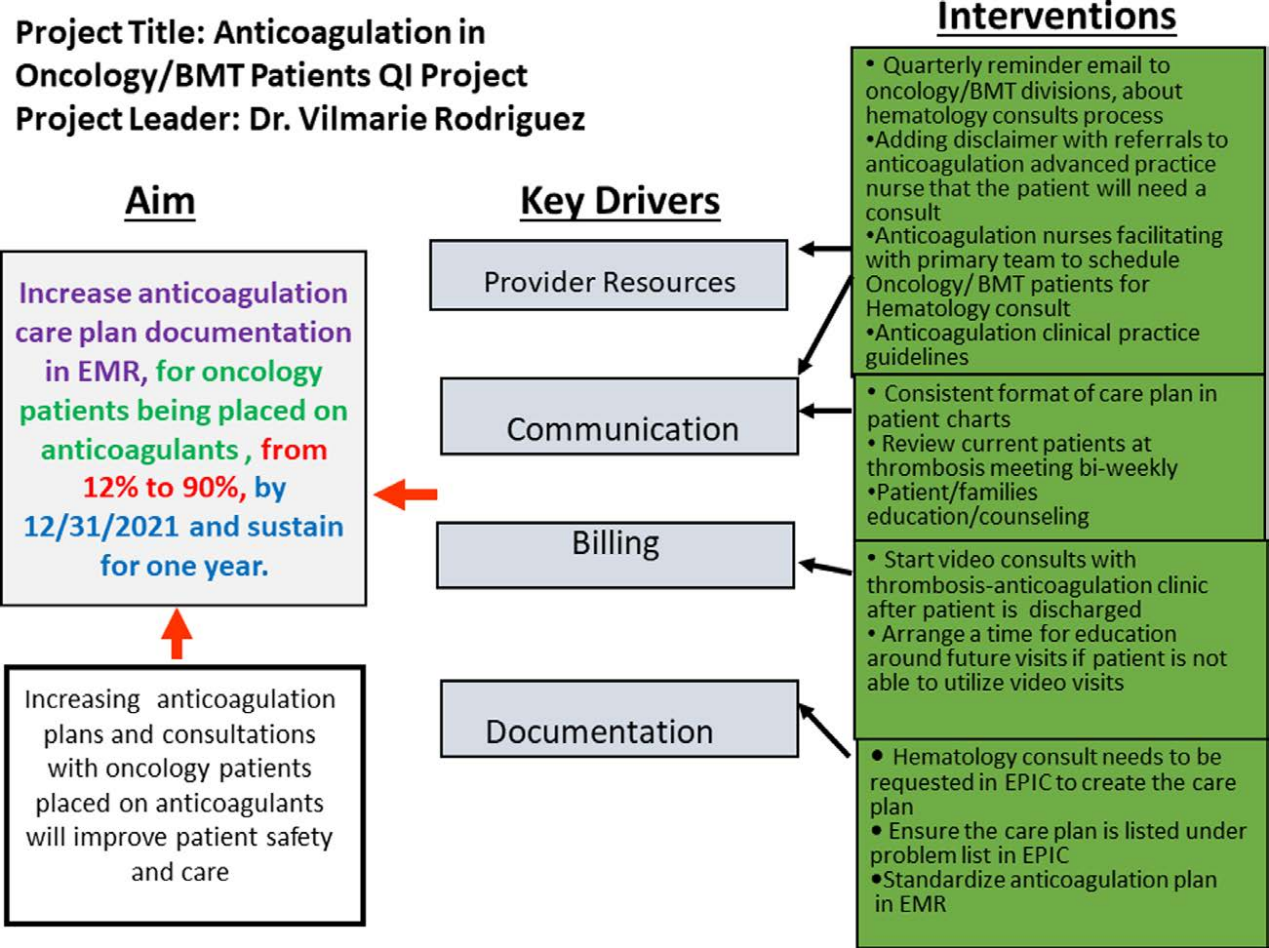
Key driver diagram.

A hemostatic assessment of each oncology patient receiving anticoagulation (eg, those with the diagnosis of VTE and those considered at risk for VTE) and documenting an individualized anticoagulation care plan were the key processes that were identified as priorities to improve anticoagulation care for patients (Fig. 1). The anticoagulation care plan provided a summary: date of thrombosis diagnosis, type of thrombosis (venous or arterial), anatomic location, provoking factors [eg, central venous catheters (CVC), infection, asparaginase chemotherapy, thrombophilia], type of anticoagulant, intensity of anticoagulation if indicated, the recommended duration of therapeutic anticoagulation, and suggested date to repeat radiological imaging studies to address thrombosis response. Two anticoagulation advanced nurse practitioners provide anticoagulation patient education and facilitate coordination of care with the assistance of three registered nurses.

Bleeding episodes during anticoagulation therapy were categorized according to the International Society on Thrombosis and Haemostasis criteria for anticoagulated patients. Briefly, major bleeding was defined as (a) fatal hemorrhage, or (b) symptomatic bleeding in a critical organ or area, or (c) bleeding causing a drop in hemoglobin level (≥20 g/L) or leading to transfusion of blood (≥2 units).^12^ Clinically relevant nonmajor bleeding (CRNMB) was defined as any sign or symptom of hemorrhage that did not meet the major bleeding criteria but did meet one or more of the following criteria: (a) requiring medical intervention by a healthcare professional, (b) leading to hospitalization or increased level of care, or (c) prompting a face-to-face evaluation.^13^ All bleeds not meeting the major bleeding or CRNMB criteria were classified as minor. Also aligned with International Society on Thrombosis and Haemostasis recommendations, symptomatic VTE recurrence was defined as any radiologically confirmed thrombus (whether noncontiguous new thrombus or progression of previously defined thrombus) accompanied by signs/symptoms corresponding to the site of VTE. VTE progression was defined as either an increase in thrombus burden or a change from nonocclusive to occlusive thrombosis. VTE progression and symptomatic recurrence while receiving anticoagulation were adjudicated by chart and imaging review by the corresponding author.^14^ Bleeding and thrombosis progression/recurrence were expected to occur in this medically complex patient cohort. A detailed review of events included (i) appropriate anticoagulation monitoring (eg, anti-Xa levels for those on enoxaparin therapy), (ii) platelet transfusions for thrombocytopenia, and (iii) interruption of anticoagulation during surgical/invasive procedures.

The target patient population included all oncology and bone marrow transplant (BMT) patients receiving anticoagulation therapy in therapeutic or prophylactic dosing. Patients were identified by: (1) automatic EPIC generated report with newly diagnosed patients with thrombosis across the hospital, (2) real-time communication with thrombosis and anticoagulation care team and oncology/BMT providers, and (3) Inpatient hematology consultations. The study team reviewed baseline data of patients with a thrombosis diagnosis served by NCH oncology, and BMT services from 1/1/2019 through 1/1/2020. Patient demographics and characteristics were reviewed with particular attention to the presence of a hematology consult (inpatient or outpatient EMR documentation), documentation of an anticoagulation care plan, appropriate duration, and monitoring (if indicated) of the anticoagulant use, bleeding, and recurrent/progressive thrombosis while on anticoagulation. Appropriate duration of anticoagulation was defined as at least 6–12 weeks or until the VTE risk factor was no longer present (eg, removal of CVL, discontinuation of asparaginase therapy). If receiving enoxaparin, monitoring was defined as at least one anti-factor Xa level per month.

### Study of Interventions and Measures

A group of stakeholders (including members of the thrombosis-anticoagulation care team and a quality expert), met several times in brainstorming sessions and identified four key drivers: provider resources, communication, billing, and documentation. Based on these, interventions were created addressing each key driver barrier (Fig. 1). These interventions were simultaneously implemented with the main goals of improving access to hematology consultations and documentation of an individualized anticoagulation care plan for each patient. A communication (eg, Situation Background Assessment Recommendations) mandating the need for hematology consultations for any newly diagnosed VTE, and high-risk patients where pharmacological prophylaxis was considered was sent to all hematology-oncology and BMT providers (physicians and nursing staff) in October 2020. The principal recommendations of this communication included mandatory hematology consults for all new VTE diagnoses or patients deemed at risk for VTE requiring pharmacologic prophylaxis, with the outpatient clinic (or telehealth) follow-up with the thrombosis team.

Access to outpatient hematology consultations was one of the barriers identified and needed improvement. Seeing these patients on the same day as their oncology/BMT clinic visit was not always possible (eg, time constraints, inability to bill for hematology services on the same day as oncology visit). The addition of telehealth visits to our hematology consultations provided an alternative for follow-up to patients/families.

During the hematology consultation (eg, inpatient, outpatient, or telehealth visit), an individualized anticoagulation action plan was developed for the patient and entered into the EMR. This plan was derived based on best practice guidelines recommended by the American Society of Hematology and the American College of Chest Physicians.^15,16^ Additionally, institutional clinical practice guidelines (CPGs) were developed and updated for (i) LMWH, (ii) warfarin, (iii) unfractionated heparin, (iv) DOACs, (v) thrombolysis, and (vi) pulmonary embolism. These CPGs were made readily available for all providers across the institution. Finally, we developed consultation note templates to provide recommendations for anticoagulation management (eg, type and dose of anticoagulant, duration, anticoagulant monitoring if indicated, peri-procedure management, recommended platelet threshold, and the approximate date and type of radiological imaging to document thrombosis response).

With the growing number of oncology/BMT patients followed by our thrombosis program, close follow-up and continuity of care of these patients was required on a regular basis. A list of oncology/BMT patients on anticoagulation is maintained securely by the anticoagulation team and regularly updated. Patient consultations and anticoagulation care plans were reviewed at each thrombosis team meeting (twice a month). If a patient was due for anticoagulation monitoring or repeat radiological imaging, our team would follow-up with patients/families and the primary oncology/BMT provider.

### Analyses

All the data were summarized using standard descriptive statistics, frequency, and percentage for qualitative variables, and median and interquartile range (IQR) for quantitative variables. Comparisons between the pre- and post-quality improvement (post-QI) cohorts were done using Fisher exact, chi-square, or Wilcoxon rank-sum tests. All *P* values were two-sided, and those less than 0.05 were considered statistically significant. Analyses were completed using SAS software, version 9.4 (SAS Institute, Cary, N.C.).

## RESULTS

### Baseline Data

Seventeen patients were identified in the baseline or pre-QI cohort (Table 1). Slightly over half were men (64.7%), with a median age (range) of 10.3 years (4.1–13.4). Most patients had the underlying diagnosis of leukemia [14 (82.4%)], and the primary indication for anticoagulation therapy was thrombosis [16 (94.1%)]. Close to half of these events were CVC-associated thrombosis involving the upper extremities [8 (47%)].

**Table 1.** Patient Demographics Summary and Comparisons Pre-QI and Post-QI

Characteristic	Pre-QI (N = 17)	Post-QI (N = 34)	*P*
**Male Sex**	11 (64.7)	18 (52.9)	0.42
**Malignancy Type**			0.0051
Leukemia	14 (82.4)	11 (32.4)	
Lymphoma	1 (5.9)	4 (11.8)	
Solid	2 (11.2)	17 (50.0)	
Brain	0 (–)	2 (5.9)	
**Age at Thrombosis**	10.3 (4.1–13.4)	14.4 (9.9–18.4)	0.0382
**Indication for Anticoagulation**			0.27
Thrombosis	16 (94.1)	27 (79.4)	
History of thrombosis	0 (–)	5 (14.7)	
Other	1 (5.9)	2 (5.9)	
**Thrombosis Type**			0.60
UE	8	12	
LE	4	5	
PE	2	5	
CSVT	3	3	
Right atrial	0	5	
SVC	0	2	
IVC	0	1	
Portal vein	0	1	
** Anticoagulant Used**			0.0126
LMWH only	12	11	
DOAC only	1	8	
LMWH + DOAC	3	15	
LMWH + warfarin	1	0	
**Consult Type**			<0.0001
None	8 (47.1)	0 (–)	
Inpatient	8 (47.1)	13 (38.2)	
Telehealth	0 (–)	12 (35.3)	
Infusion	0 (–)	5 (14.7)	
Clinic	1 (5.9)	4 (11.8)	
**Anticoagulation Plan and Consult**	2 (11.8)	34 (100)	<0.0001
**Anticoagulation Plan**	7 (43.8)	34 (100)	<0.0001
**Duration of Therapeutic Anticoagulation (d**)	164 (79–314)	205 (94–288)	0.60
**Duration of Prophylactic Anticoagulation (d**)	289 (179–658)	121 (58–304)	0.09
**Appropriate Monitoring (enoxaparin**)	8 (47.1)	34 (100)	<0.0001
**Bleeding**			
CRNMB	5 (29.4)	11 (34.4)	0.99
Major bleed	1 (5.9)	1 (2.9)	0.99
Minor bleed	0	2 (5.9)	0.55
**Recurrent/Progressive VTE**	2 (11.8)	5 (14.7)	0.99

*Note 4 post-QI patients still on therapeutic AC and 14 still on prophylaxis as of late September 2022.

UE, upper extremity; LE, lower extremity; PE, pulmonary embolism; CSVT, cerebral sinus thrombosis; superior vena cava thrombosis; inferior vena cava thrombosis; CRNMB, clinically relevant nonmajor bleeding; VTE, venous thromboembolism.

Pre-QI, nine patients had a hematology consultation completed (52.9%), and 7 had an anticoagulation plan documented (43.8%) (Table 1). Only two patients had a hematology consult and anticoagulation care plan documented (11.8%). Appropriate monitoring of anticoagulation (as indicated) was provided in nearly half of the patients.

### Interventions, Process, and Outcome Measures

**Fig. 2.**
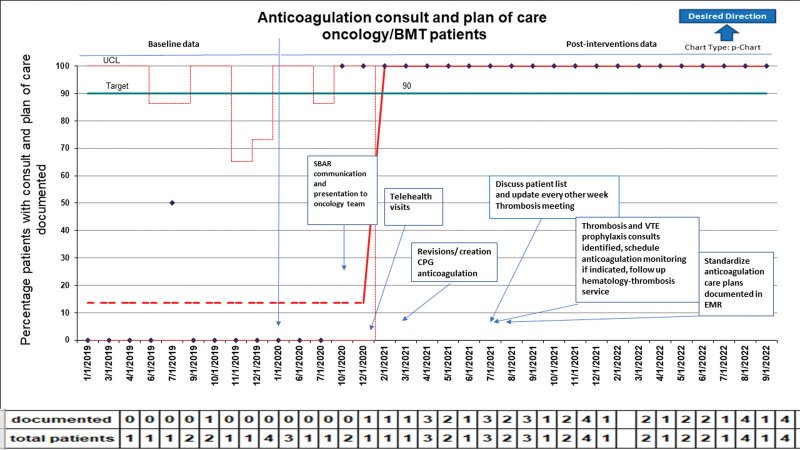
p-Chart illustrating the percentage of patients with consults and anticoagulation care plan of care documented. Broken red line: baseline data. Arrow indicates time first PDSA with interventions implemented sequentially. Green line indicates target goal.

Following interventions according to our key driver diagram, we observed a significant improvement in hematology consultations and of an anticoagulation care plan in the post-QI cohort (Table 1). Similarly, we observed an improvement in anticoagulation monitoring (eg, enoxaparin). Figure 2 illustrates the p-chart diagram with annotated interventions. The availability and flexibility of telehealth visits for hematology consultations resulted in improved access to our services (37.5% of all consultations; Table 1).

**Fig. 3.**
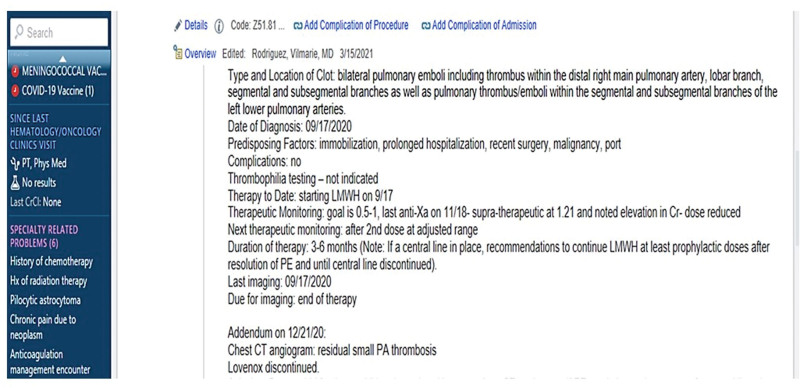
Example of an anticoagulation care plan.

Thirty-four patients comprised the post-QI cohort represented primarily by solid tumors (50%) and hematological malignancies (leukemia and lymphoma 44%). All oncology/BMT patients requiring anticoagulation were seen by hematology (n = 34), and each patient had an individualized anticoagulation care plan (Table 1). Once the consultation was completed, the hematology provider or anticoagulation advanced nursing provider completed an anticoagulation care plan listed under the problem list on the patient’s EMR (Fig. 3). The Oncology/BMT providers’ satisfaction with consultations were not forrmally measured.

Anticoagulation consults and documentation of care plans improved above our target goal to 100% (n = 34) and were sustained for nearly 2 years (Fig. 2). Anticoagulation monitoring for those patients treated with enoxaparin was significantly improved post-QI [pre-QI 47.1% (n = 8) to post-QI 100% (n = 34); *P* < 0.0001] (Table 1). Clinical outcome measures, including bleeding and thrombosis recurrence/progression, were not significantly different pre- and post-QI (Table 1). Pre-QI bleeding events included 5 (29.4%) CRNMB [three epistaxis (n = 3), hematemesis (n = 2); all events were supported with platelet transfusions] and one (5.8%) major bleeding (intraabdominal hematoma requiring interventional radiology drainage). Post-QI bleeding events included 11 (32.3%) CRNMB all supported with platelet transfusions [epistaxis (n = 5), hematemesis (n = 1), one hemoptysis (n = 1), menses breakthrough bleeding (n = 2), postoperative wound bleed (n = 1), hemorrhoids bleed (n = 1)] and one (2.9%) major bleeding (postoperative hematoma requiring surgical intervention). Thrombosis events pre-QI (ALL relapsed disease with previous history of DVT n = 2); post-QI (ALL noncompliant with anticoagulation therapy recurrent disease n = 1, ALL with prior history of superficial upper extremity thrombosis n = 1, Wilms tumor postsurgical n = 1, history of DVT and neuroblastoma with history of multiple CVLs n = 1, recurrent metastatic disease n = 1).

## DISCUSSION

Through use of QI methodology, our thrombosis-anticoagulation care team has become actively involved in the anticoagulation management of oncology patients and has effectively engaged patients/families with their anticoagulation care needs and education. We successfully initiated interventions to improve communication with oncology/BMT providers and improved access to our services. Anticoagulation care clinical pathway guidelines and patient educational materials simultaneously provided a comprehensive and standardized approach to anticoagulation management to a medically complex patient population. Documentation of an individualized anticoagulation care plan in the EMR, provided an effective way to keep our team and primary oncology/BMT providers, with an up-to-date summary of thrombosis and anticoagulation indications, as well as details of antithrombotic management. Unfortunately, a formal assessment of oncology/BMT providers’ satisfaction to our hematology consultations and documentation of an individualized care plan for each patient was not completed as part of this QI project.

Venous thromboembolism continues to be a rising complication in pediatrics. A retrospective study using the Pediatric Health Information System database from 2008 to 2019 showed an alarming rise in VTE from 46 VTE cases per 10,000 admissions in 2005 to 106 cases per 10,000 admissions in 2013, translating into a 130% increase.^17^ Most patients had a chronic health condition including cancer. A population-based study in the United Kingdom showed a VTE rate of 1.52 per 1000 person-years in children with cancer versus 0.06 per 1000 person-years in the control group of children without cancer [HR 28.3; 95% CI, 7.0–114.5].^18^ Thrombosis rates vary among the different pediatric malignancies with 1%–36% in ALL, 7%–16% in soft tissue sarcoma and less than 1%–2.8% in brain tumors.^19–24^ The underlying risk factors for VTE in pediatric cancer are multifactorial and include the use of CVC, chemotherapy, surgery, thrombophilia, and oncologic disease-related factors (eg, mediastinal mass, leukocytosis). The majority of thrombotic events are CVC associated with a reported symptomatic venous-catheter-associated VTE of 2.6%–36.7% and asymptomatic VTE rates of 5.9%–43%.^25–27^ Similar to the published literature, thrombosis complications in our study were most frequently encountered in leukemia and lymphoma groups followed by solid tumors (Table 1).

Thromboprophylaxis guidelines are available for adults with cancer.^3,10^ Unfortunately, similar guidelines for anticoagulation management in pediatric oncology are not available.^15,16^ Consequently, several recommendations provided for antithrombotic therapy in pediatric oncology are extrapolated from the adult literature.^28,29^ Following the United States Food and Drug Administration pediatric approval for the use of rivaroxaban and dabigatran for primary and secondary VTE prevention, more pediatric medical providers are prescribing these agents for the therapy of acute VTE rather than the use of conventional anticoagulation such as low molecular weight heparin or warfarin. Knowledge on the use of these agents (eg, direct oral anticoagulants-DOACs), pharmacokinetics, contraindications for their use, and potential drug interactions are required to provide safe anticoagulation management.^30,31^ Limited data are available regarding the safety and efficacy of DOACs among pediatric cancer patients. After Food And Drug Administration approval, and given their ease of administration, predictable pharmacokinetics, lack of food interactions, we noted a significant increase in DOAC use during the study period (Table 1). Efficacy and safety outcomes of DOAC use in this cohort are being investigated through a multicenter institutional review board-approved study and will be reported in a follow-up manuscript. Whether conventional anticoagulation or DOACs are used, careful monitoring of anticoagulation care (including close monitoring of platelet blood counts to mitigate risks for bleeding), appropriate interruption of therapy before invasive procedures, and following the recommended duration of primary and secondary VTE treatment and prevention are essential order to provide patients with optimal thrombosis outcomes. Given our small sample size we were unable to document and improvement in clinical outcomes in the current study, and hope to report them in a follow-up publication. In our study, measures on anticoagulation quality such as appropriate monitoring for those patients receiving enoxaparin, significantly improved compared with baseline data supporting the need to involve hematologists and an anticoagulation care team with expertise in thrombosis management in the anticoagulation management of the pediatric cancer patient.

Access to hematology consults was one key driver identified to improve access to consultations. The use of telehealth visits during the COVID-19 pandemic allowed many medical practices to continue offering services to patients without the risk of viral exposure in the clinical or hospital setting.^32^ This intervention provided slightly over one-third of consultations in addition to facilitating follow-up after anticoagulation initiation.

Our study has several limitations. We are a large pediatric hospital with a strong patient safety and QI program. Several interventions were simultaneously created and implemented, and the complementary contributions of each one of them might have led to the rapid achievement of the target goal and its sustainability. It is possible some of the achievements toward our specific aim were accomplished early on due to the rapid spread of our interventions and available resources in our institution (eg, dedicated anticoagulation care nurses, clinical pathway guidelines program, pharmacy). These resources might not be present in other institutions with fewer overall resources, making achieving similar results more challenging. Most of our interventions focused on communication, education, and team collaboration. Having a formal anticoagulation care team program served as a “safety net” reminding referring oncology/BMT providers of the need for consultations and follow up with our team. In some instances, our team facilitated the referral process to our services from primary oncology/BMT providers. Despite an improvement in process measures, clinical outcome measures (bleeding and recurrent thrombotic events) were not significantly different pre- and following QI interventions. This may be secondary to the small sample size of the current work, and we anticipate an improvement in the outcome measures as we continue to follow this cohort. A standardized approach to antithrombotic therapy led by a dedicated anticoagulation team has previously shown improved clinical outcomes in pediatric cardiology, stroke, and inflammatory bowel disease.^33–35^

Other measures of compliance with the anticoagulation care plan such as correct dosing and duration of anticoagulants, compliance with recommended radiological imaging for follow-up, and adherence to recommended platelet transfusions can be further studied to address the degree of anticoagulation management standardization in our oncology patients.

### Conclusions

In conclusion, provision of anticoagulation consults and documentation of an individualized care plan for pediatric oncology/BMT patients was improved through this QI initiative. Communication, education, and improved access to care addressed barriers to patient referral to our services and improved documentation of an individualized anticoagulation care plan. QI methodology not facilitated achieving of our study aim but also ensured sustainability of our efforts. Improvement in clinical outcome measures is anticipated with continued follow-up of this cohort.

## DISCLOSURE

The authors have no financial interest to declare in relation to the content of this article.

## ACKNOWLEDGMENTS

We thank patients and families for allowing our Thrombosis and Anticoagulation Team to participate in their care.
